# Increased histone citrullination in juvenile idiopathic arthritis

**DOI:** 10.3389/fmed.2022.971121

**Published:** 2022-08-19

**Authors:** Zuzana Parackova, Irena Zentsova, Hana Malcova, Dita Cebecauerova, Anna Sediva, Rudolf Horvath

**Affiliations:** ^1^Department of Immunology, 2nd Faculty of Medicine, Charles University, University Hospital Motol, Prague, Czechia; ^2^Department of Paediatric and Adult Rheumatology, University Hospital Motol, Prague, Czechia

**Keywords:** juvenile idiopathic arthritis, citrullination, histone, neutrophil, NETosis, peptidyl arginine deiminases (PAD), carbamylation

## Abstract

**Objective:**

Posttranslational modifications (PTMs) of proteins are crucial for regulating various biological processes. However, protein alteration *via* PTMs, and consequently, the creation of new epitopes, can induce abnormal autoimmune responses in predisposed individuals. Immunopathogenesis of several rheumatic diseases, including the most common childhood form, juvenile idiopathic arthritis (JIA), is associated with the generation of autoantibodies against such modified proteins. Dysregulated generation of neutrophil extracellular traps (NETs) can be a source of post-translationally altered proteins. Thus, we investigated the role of PTMs and the presence of NET-associated markers in JIA patients.

**Methods:**

We recruited 30 pediatric patients with JIA (20 with active disease and 10 in remission) and 30 healthy donors. The serum concentrations of citrullinated histone H3 (citH3), peptidyl arginine deiminases (PADs), and NET-related products were detected using ELISA, and the number of citH3+ neutrophils was assessed using flow cytometry.

**Results:**

The serum levels of citH3 and PADs were higher in active as well as in remission JIA patients than in healthy donors. Similarly, the number of citH3+ neutrophils was higher in the peripheral blood of patients with JIA, implying an enhanced process of NETosis. This was effectively reflected by elevated serum levels of NET-associated products, such as neutrophil elastase, LL37, and cell-free DNA-histone complexes. Additionally, 16.7% of active JIA patients were seropositive for carbamylated autoantibodies, the levels of which declined sharply after initiation of anti-TNFα therapy.

**Conclusion:**

Collectively, our data suggest that the accelerated process of NETosis and PTMs in JIA may result in the generation of anti-citrullinated/carbamylated autoantibodies against various epitopes later in life, which could be prevented by effectively regulating inflammation using immune therapy.

## Significance and innovations

- Increased NETosis might contribute to disease immunopathology.
- Enhanced PTMs in JIA may result in the generation of anti-citrullinated/carbamylated autoantibodies later in life.

## Introduction

Posttranslational modifications (PTMs) of proteins are crucial for protein regulation, folding, intracellular trafficking, interactions with ligands or other proteins, and eventually, changes in immunogenicity. However, protein alterations *via* PTMs, and consequently, the creation of new epitopes, can induce an abnormal autoimmune response in predisposed individuals. Juvenile idiopathic arthritis (JIA) is the most common inflammatory rheumatic joint disease among children, characterized by joint inflammation of an unknown origin that persists longer than 6 weeks and occurs before the age of 16 (1–3). Immunopathology of several rheumatic diseases, including JIA, is associated with the generation of autoantibodies against such modified proteins (4–9).

Citrullination is the best characterized PTM in the field of rheumatology, with antibodies against cyclic citrullinated peptides (ACPA) being the gold standard for the diagnosis and classification of patients with rheumatoid arthritis (RA) (5). Similar to that for adult RA, ACPA is detected in patients with RF+ (Rheumatoid Factor Positive) polyarticular JIA; higher ACPA levels are correlated with a greater risk of more aggressive and erosive disease (1). Recent evidence suggests that along with citrullination, other PTMs such as carbamylation can trigger an autoimmune response (10, 11). Carbamylation is the chemical conversion of lysine into uncharged homocitrulline within the polypeptide, usually resulting in protein loss-of-function. *In vivo* carbamylation is mainly dependent on myeloperoxidase (MPO) activity, an enzyme released upon neutrophil activation. Thus, the level of carbamylation is enhanced in an inflammatory environment. Homocitrulline residues may act as neoepitopes, leading to the production of autoantibodies as previously described in RA and JIA (6, 12). Hence, PTMs may elicit the production of a wide spectrum of autoantibodies, highlighting their diagnostic and prognostic roles in clinical practice for patients with JIA.

Activated neutrophils extrude web-like structures composed of decondensed DNA together with cytosolic and granule proteins, called neutrophil extracellular traps (NETs), to trap and kill bacteria (13–15). NETs are highly biologically active structures and powerful initiators of inflammation (13), because they contain various proteases, such as proteinase 3, neutrophil elastase (NE) and MPO (16, 17). This classic antimicrobial defense mechanism is also implicated in the pathogenesis of several inflammatory rheumatic diseases. NETs can be a source of autoantigens and can escalate inflammation directly or indirectly by modulating and activating other immune cells (18–22). Enhanced NET-related products were observed in the synovial fluid and peripheral blood of patients with RA (18, 23–25). Moreover, Khandpur et al. showed that NET associated antigens may serve as a target for ACPA generation as a result of externalization of citrullinated autoantigens by neutrophils during NETosis (23).

Importantly, the enzyme peptidyl arginine deiminase (PAD), involved in protein citrullination, also participates in the initial steps of NET formation by converting arginyl residues in histone to citrulline, reducing their charge-based interaction with DNA, and promoting DNA decondensation (26). PAD2 and PAD4 are enriched in neutrophils, released during NETosis or cell death and likely drive citrullination under inflammatory conditions (27, 28). Even though PAD4 is generally located intracellularly, healthy viable neutrophils have also active PAD4 exposed on their surface and spontaneously secrete PAD2 (29), contributing to enhanced NETosis and citrullination. We surmised that dysregulated PTMs and NETosis may be implicated in the pathogenesis of JIA. Therefore, in this study, we assessed the role of PTMs and NETosis-associated products in JIA.

## Patients and methods

### Patients

The biologic material was obtained from patients were followed at the Department of Pediatric and Adult Rheumatology, University Hospital in Motol and fulfilled the International League of Association for Rheumatology (ILAR) criteria. We included 30 pediatric JIA patients (33.3% male; mean age 13.1 ± 3.7; JADAS71 8.43 ± 7.26) and 30 healthy donors (30% male; mean age 16.61 ± 9.88 years) in the study. Of the 30 JIA patients, 20 had active disease (juvenile arthritis disease activity score; JADAS71 ≥ 1; mean 12.65 ± 4.94) and 10 were in remission (JADAS71 <1 lasting for at least 6 months; mean (0). JADAS is constructed around four elements: (1) the active joint count, (2) physician global assessment, (3) parent/patient visual analog scale (VAS) of wellbeing and (4) erythrocyte sedimentation rate (ESR) (30). Both groups contained patients with oligoarticular/oligoarticular extended, polyarticular and enthesis-related arthritis (ERA) JIA subtypes. All patients included in JIA active group consequently were treated with TNF inhibitors (TNFi). Experiments in JIA active group were performed before the TNFi therapy initiation. The healthy donors had no prior history of autoimmune disease. Not all patients were involved in all experiments owing to the limited amount of blood available per participant. Demographic characteristics of the patients are summarized in Supplementary Table 1.

Written informed consent was obtained from all the patients or the patients' parents/guardians in accordance with the Declaration of Helsinki, and the study was approved by the Ethics Committee of University Hospital Motol.

### ELISAs

ELISAs were performed to measure the serum concentrations of MPO, NE (Abcam, Cambridge, UK), LL-37 (Hycult Biotech, Wayne, USA), DNA-histone complexes (Merck Millipore, Burlington, USA), citrullinated histone H3 and PAD type 4 (Cayman Chemicals, Ann Arbor, USA) and type 2 (LSBio, Seattle, WA, USA), and carbamylated autoantibodies (MyBioSource, San Diego, USA) according to the manufacturers' instructions.

### NETting neutrophil detection

NETting neutrophils were analyzed according a previously published study (31). Briefly, peripheral blood was stained with a mixture of antibodies containing anti-lineage specific markers (CD3 clone MEM-57, CD19 clone LT19, CD20 clone LT20, CD56 clone MEM-188, CCR3 clone 5E8) conjugated with FITC (Exbio, Prague, Czech Republic) to exclude T, B and NK cells, CD66b-PC7 (clone G10F5), CD16-A700 (clone 3G8) (Biolegend, San Diego, CA, USA) to define neutrophils as Lin-CD66b+CD16+ and exclude eosinophils and basophils, MPO-PE (Exbio) and citH3-A647 (Abcam) for 20 min in the dark, at room temperature and then hypotonically lysed. Samples were acquired on a BD Fortessa (BD Biosciences, San Jose, USA) and analyzed using FlowJo software.

### Statistics

Data are presented as the median of values obtained from at least four independent experiments. Statistical analysis was performed using non-parametric one-way analysis of variance (ANOVA) with multiple comparison Dunn's post-test where applicable. A two-tailed paired Wilcoxon or unpaired Mann–Whitney *t*-test was also applied for data analysis using GraphPad Prism 8. Values of *p* < 0.05 (^*^), *p* < 0.01 (^**^), *p* < 0.001 (^***^) and *p* < 0.0001 (^****^) were considered statistically significant.

## Results

### Increased levels of citrullinated histone H3 in patients with JIA

**Figure 1:**
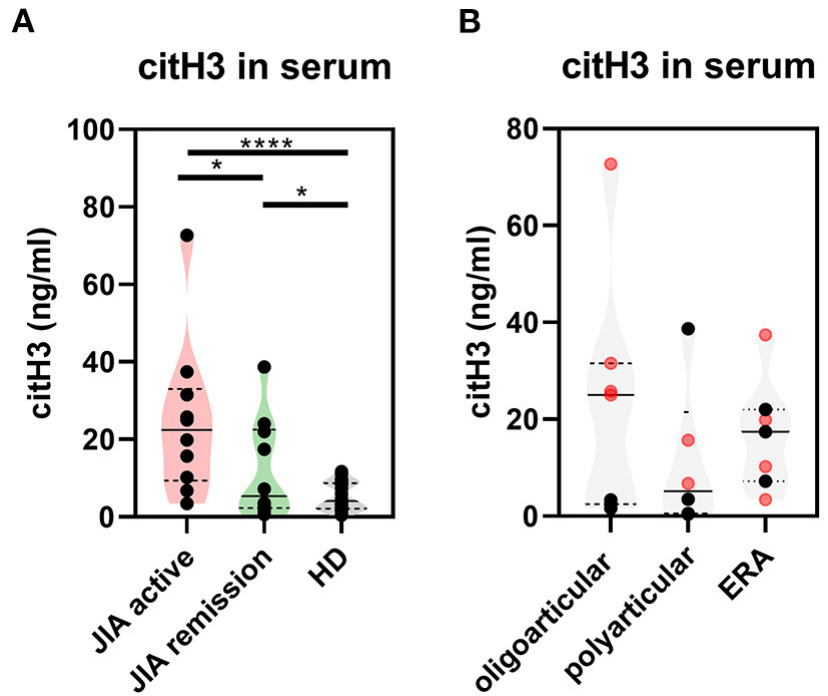
Citrullinated histone H3 in JIA. **(A)** Serum levels of citrullinated histone H3 (citH3) in JIA active (*n* = 10), JIA remission (*n* = 10), and healthy donors (*n* = 26) were detected using ELISA. **(B)** citH3 levels were compared between the groups. Patients with JIA active (in red) and JIA remission (in black) were divided, according to JIA subtypes, into oligoarticular/oligo-extended, polyarticular, and ERA (enthesis-related arthritis) groups. Values are standardized and expressed as median values. Statistical analyses were performed using unpaired *t*-tests. Values of *p* < 0.05 (*), *p* < 0.01 (**), *p* < 0.001 (***), and *p* < 0.0001 (****) were considered statistically significant.

To determine whether NETs are generated in JIA, we examined serum from JIA patients (*n* = 30) (Supplementary Table 1) for presence of NETosis markers: cell-free DNA (DNA-histone complexes), myeloperoxidase (MPO), neutrophil elastase, LL37 and citrullinated histone H3 (citH3) relative to healthy donors. The serum levels of citrullinated histone H3 (citH3) were significantly higher in both the active and remission JIA groups than in healthy donors (HD) (Figure 1A). Interestingly, we observed clear differences in citH3 concentrations between the oligoarticular/oligo-extended subgroups, but not the polyarticular or enthesis-related JIA (ERA) JIA subgroups, of the active and remission JIA groups. The higher levels were more characteristic for patients with the active state of the disease than those in remission (Figure 1B). This observation implies an increased NETosis and citrullination in patients with JIA.

### Elevated levels of citrullination-associated enzymes in patients with JIA

**Figure 2:**
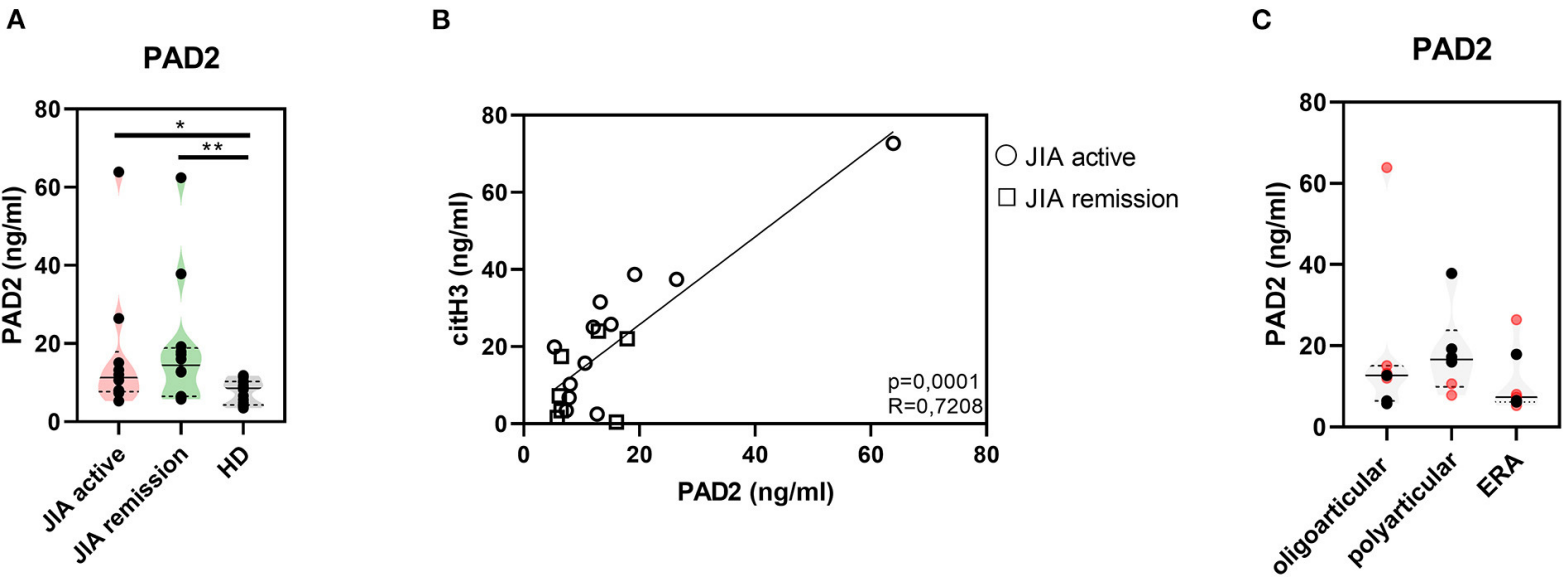
Peptidyl arginine deiminase 2. **(A)** Peptidyl arginine deiminase 2 (PAD2) concentrations in JIA active (*n* = 10), JIA remission (*n* = 10), and healthy donors (HD = 16) detected using ELISA. **(B)** Relationship between the PAD2 and citH3 levels in JIA active and JIA remission groups. **(C)** PAD2 levels in JIA subtypes: oligoarticular/oligo-extended, polyarticular, and ERA groups. Patients with active JIA are displayed in red and patients with JIA remission are shown in black. Values are standardized and expressed as median values. Statistical analyses were performed using unpaired *t*-tests. Values of *p* < 0.05 (*), *p* < 0.01 (**), *p* < 0.001 (***), and *p* < 0.0001 (****) were considered statistically significant.

**Figure 3:**
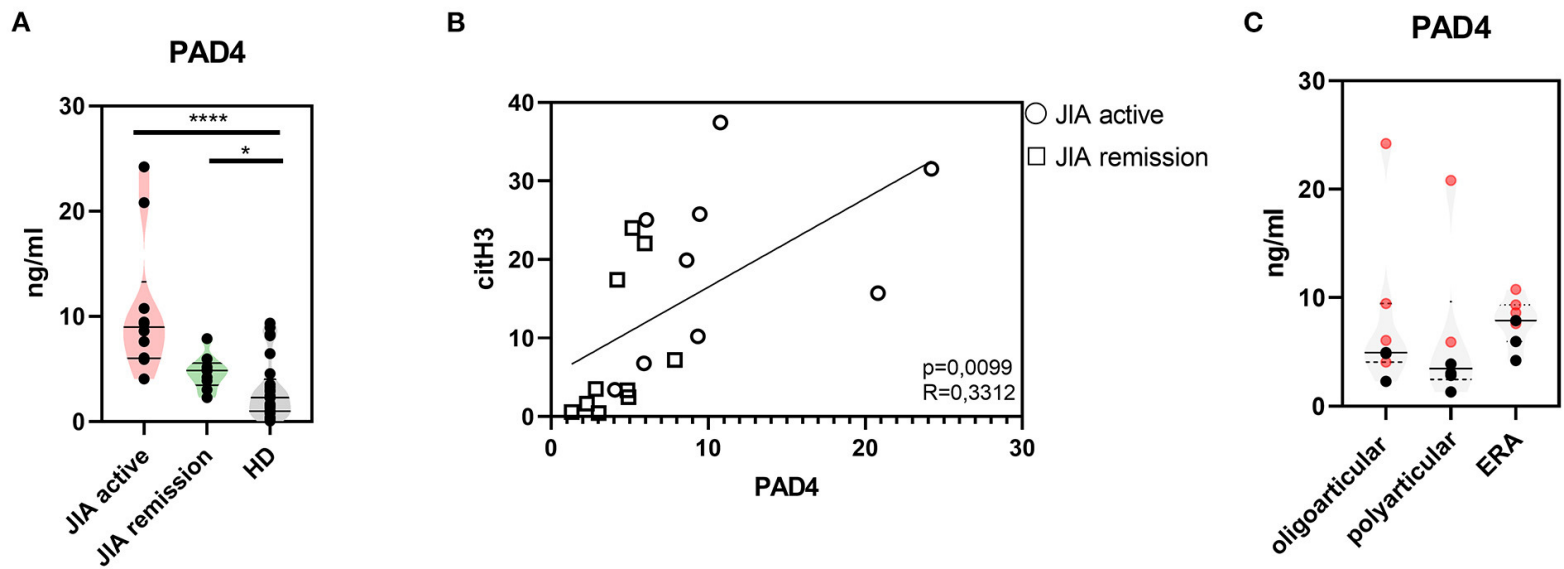
Peptidyl arginine deiminase 4. **(A)** Peptidyl arginine deiminase 4 (PAD4) concentrations in JIA active (*n* = 10), JIA remission (*n* = 10), and healthy donors (HD = 16) detected using ELISA. **(B)** Correlations between the PAD4 and citH3 levels in JIA active and JIA remission groups. **(C)** PAD4 levels in JIA subtypes: oligoarticular/oligo-extended, polyarticular, and ERA groups. Patients with active JIA are displayed in red and patients with JIA remission are shown in black. Values are standardized and expressed as median values. Statistical analyses were performed using unpaired *t*-tests. Values of *p* < 0.05 (*), *p* < 0.01 (**), *p* < 0.001 (***), and *p* < 0.0001 (****) were considered statistically significant.

Further, to study citrullination in JIA, we assessed serum concentrations of PAD 2 and 4, the two main enzymes implicated in the protein citrullination process (Figures 2A, 3A). Compared with the HD, the active and remission JIA groups displayed substantially increased the concentrations of PAD2 and 4; their concentrations positively correlated with citH3 serum levels (Figures 2, 3). Similar to citH3 levels, we noted increased levels of PAD4, but not PAD2 levels, between the oligoarticular/oligo-extended JIA subgroups of the active and remission groups (Figures 2B, 3B).

### Expression of citrullinated histone on surface of neutrophils in patients with JIA

**Figure 4:**
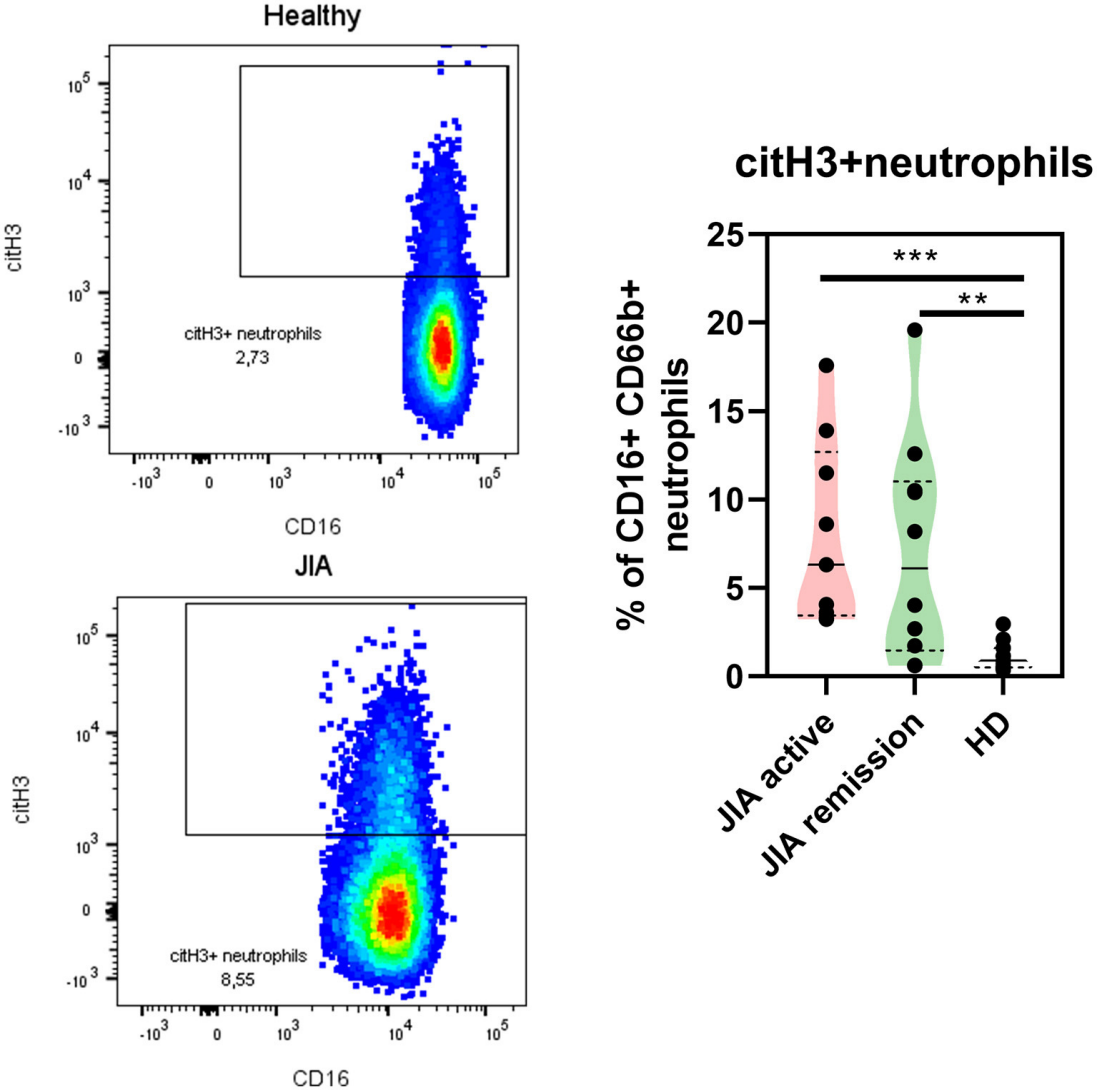
NETting neutrophils in JIA patients. Representative dot plots of the citH3+ neutrophil population (Lin-CD66b+CD16+) and quantification of citH3+neutrophils in JIA active (*n* = 9), JIA remission (*n* = 10), and healthy donors (*n* = 11) using flow cytometry. Values are standardized and expressed as median values. Statistical analyses were performed using unpaired *t*-tests. Values of *p* < 0.05 (*), *p* < 0.01 (**), *p* < 0.001 (***), and *p* < 0.0001 (****) were considered statistically significant.

Next, we explored whether NETs were present in the patients with JIA by detecting NETting neutrophils (defined as CD66b + CD16 + MPO + citH3+) in the patient and HDs periphery using flow cytometry. Even though the MPO expression on the neutrophil surface was undetectable, we noted significant changes in the citH3 expression on the neutrophil surface between healthy and patient neutrophils. Compared with the HD, the active and remission groups exhibited a significantly higher expression of citH3 (Figure 4), further providing evidence of the elevation of histone citrullination and in JIA. There were no differences between patient with the active state of the disease and the remission group.

**Figure 5:**
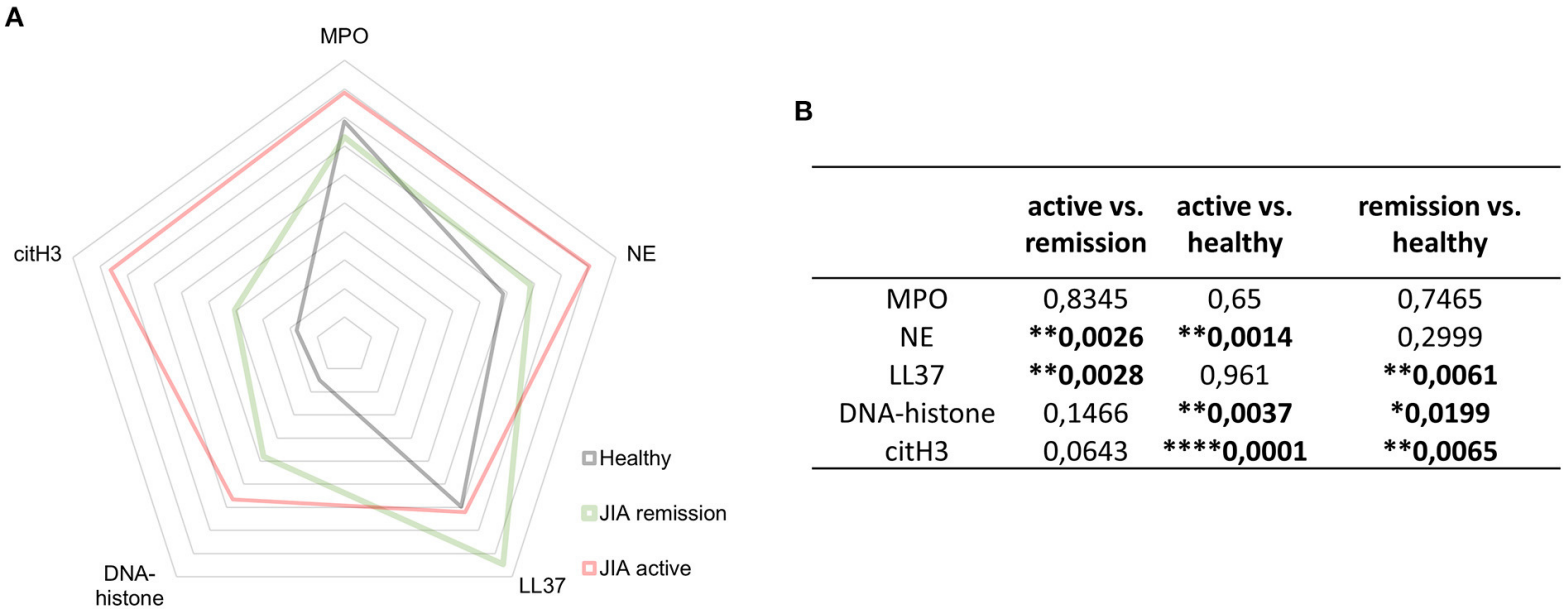
NETosis- related products. **(A)** Detection of NET-related products myeloperoxidase (MPO), neutrophil elastase (NE), LL37, DNA-histone complexes, and citH3 in JIA active (*n* = 10), JIA remission (*n* = 10), and healthy donors (*n* = 26) using ELISA. **(B)** Table containing p-values related to NET-associated products in JIA active (*n* = 10), JIA remission (*n* = 10), and healthy donors (*n* = 26) detected using ELISAs. Values are standardized and expressed as median values. Statistical analyses were performed using unpaired *t*-tests. Values of *p* < 0.05 (*), *p* < 0.01 (**), *p* < 0.001 (***), and *p* < 0.0001 (****) were considered statistically significant.

We were intrigued why we did not detect any MPO levels on neutrophil surface. In HD it might be due to low numbers of NETting neutrophils, but in JIA neutrophils with higher citH3 expression suggests increased NETosis. A possible explanation might be enhanced degranulation of JIA neutrophils, but we did not observe any differences in MPO serum levels between HD and JIA patients (Figures 5A,B). However, analyzing gene expression profiles of JIA patients in active state and in remission nanoSTRING technology, we noticed significantly lower level of MPO mRNA in JIA samples (data not shown), implying a lower content of MPO in JIA neutrophils and a possible explanation of no expression of MPO on the NETting neutrophils surface. Our data suggest an early manifestation of enhanced citrullination, potentially resulting in the generation of anti-citrullinated autoantibodies against various epitopes besides the standard ACPAs.

### Elevated NETosis-associated products in patients with JIA

In patients with JIA, citH3 was not the only elevated NETosis marker. When investigating NETosis markers in-depth, we detected significantly elevated levels of NE and DNA-histone complexes in the active JIA group compared with those in the HD. Moreover, LL37 and DNA-histone complexes were higher in the remission group than in the HD group (Figures 5A,B). These findings suggest that NETs may be involved in the pathogenesis of JIA.

### Presence of carbamylated autoantibodies in patients with JIA

**Figure 6:**
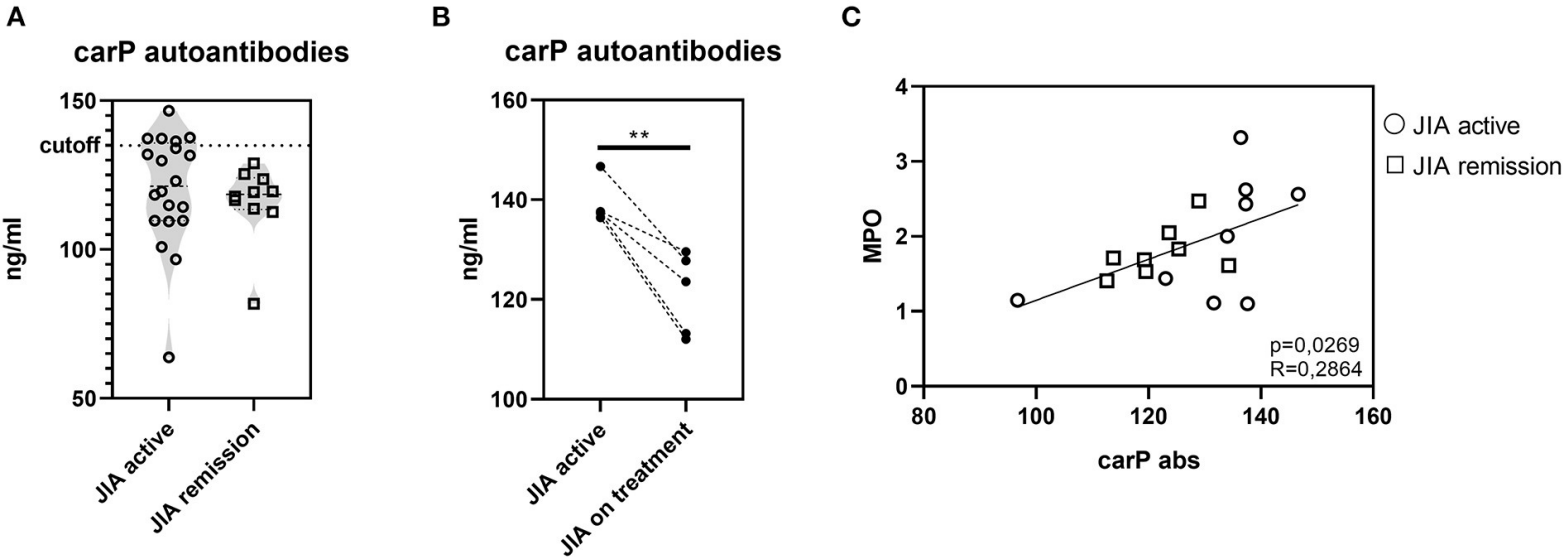
Carbamylation in JIA. **(A)** Carbamylated autoantibodies in JIA active (*n* = 20) and JIA remission (*n* = 10) detected using ELISA. **(B)** Carbamylated autoantibodies in carP seropositive patients with active JIA (*n* = 5) before and after three months of anti-TNFα treatment. **(C)** Correlation between carP autoantibodies and MPO. Values are standardized and expressed as median values. Statistical analyses were performed using paired *t*-tests. Values of *p* < 0.05 (*), *p* < 0.01 (**), *p* < 0.001 (***), and *p* < 0.0001 (****) were considered statistically significant.

To expand our investigation of PTMs and to determine whether PTMs in JIA are only limited to citrullination, we evaluated the presence of autoantibodies against carbamylated proteins (carP) (Figure 6A). Of the 30 patients with JIA, 5 (16.66%) patients, who had active JIA (two polyarticular, two oligo-extended, and one ERA), were positive for the presence of carP autoantibodies. Interestingly, examination of the kinetics of carP antibody concentrations in carP-positive patients revealed a significant decrease following a three-month treatment with anti-tumor necrosis factor-alpha (TNFα) agents (Figure 6B). This observation indicates that active inflammation may enhance carbamylation. Furthermore, cessation of inflammation leads to the reduction of PTMs. Underlying mechanisms may be dependent on MPO levels (32). Although we did not observe a significant increase in MPO levels in our cohort, MPO was slightly suppressed following anti-TNFα treatment. Moreover, carP antibodies and MPO serum concentrations positively correlated (Figure 6C), implying the importance of reducing MPO levels for a successful anti-inflammatory treatment.

## Discussion

In this brief report, we demonstrate an enhancement of citrullination in patients with JIA due to elevated concentrations of citrullinated histone H3 (citH3) and the enzymes responsible for citrullination, namely PAD2 and PAD4. Moreover, we demonstrated the elevation of other indirect serum biomarkers of NETosis in JIA such as the enzyme NE, cell-free DNA-histone complexes, and the active form of the antimicrobial peptide cathelicidin, LL37.

Citrullination is a process accompanied by a change in electrostatic charge, affecting the folding state and functioning of proteins (33). Detecting citrullination and the immune response to citrullinated proteins is imperative for aiding the early serological diagnosis of rheumatoid arthritis and gaining a better understanding of its pathophysiology. At first the main focus was on the antibodies directed to citrullinated proteins (34), but now it is realized citrullinating enzymes and citrullinated proteins may have important roles in the maintenance of the inflammatory processes in the joints. There is also accumulating evidence for a direct role of citrullination in tissue destruction in the rheumatoid synovium (35–37) For example, Sokolove et al. showed that citrullinated proteins can activate macrophages by binding to ACPA (38). Here, we report increased levels of PAD2 and PAD4 enzymes in the peripheral blood of patients with JIA, which was effectively reflected by elevated serum levels of citH3.

Neutrophils are considered to be one of the main sources of PAD enzymes in autoimmune diseases, including RA and SLE. Furthermore, neutrophils are able to express PAD4 on their surface and spontaneously produce PAD2 into their environment even in the absence of inflammatory stimuli (29, 39, 40). Those processes are independent of NETosis and contribute to extracellular protein citrullination. Histone H3 can be citrullinated by intra- as well as extracellular PADs (29). We observed a relationship between PADs and citH3 levels in the patient serum, implying that an extracellular citrullination of the histone in JIA patient is plausible. Interestingly, increased levels of PAD and citH3 levels were detected in patients with active disease and not in those in remission, supporting the importance of successful anti-inflammatory treatment in reducing levels of PAD4 and citH3. This observation implies increased citrullination in patients with JIA and a possible importance of extracellular activity of PAD enzymes, similar to those in other rheumatic diseases (18, 23). Regarding JIA patients, a study conducted by Hu et al. described a higher levels of extracellular histones in systemic-onset juvenile JIA with active state of the disease but not in the remission group (41). However, they detected DNA-histone complexes and not their citrullinated modifications as we did in this study.

A remarkable phenomenon that links citrullination to inflammation and autoimmunity is the formation of NETs. The process of NETosis is dependent on citrullination. Neutrophils release web-like structures of chromatin containing citrullinated histones, embedded with granule-derived proteins. Moreover, NET-associated proteins serve as a potential autoantigens (23, 42). We assumed that the dysregulated process of NETosis is also relevant in patients with JIA because we detected the increased NET-related products and elevated levels of neutrophils expressing citH3. Besides, PAD4, which we also found to be increased in JIA patients, is involved in the signaling pathway leading to NETosis through chromatin decondensation by histone citrullination which occurs in the nuclei (43). Elevated expression of citH3 on neutrophil surface supports the observation of elevated NETosis in JIA patients Increased NET-associated products are implicated in rheumatic diseases, as well as other autoimmune diseases, such as type 1 diabetes (16). The level of surface PAD4 was not increased on circulating neutrophils from RA and SLE patients, however it remains plausible that PAD4 expression in increased on neutrophils in site od inflammation, such as synovial fluid (44). We did not analyse the expression of PAD4 on neutrophils in JIA patients, however it is possible that similar mechanisms occur also in this pediatric pathology.

LL37 is a common component of NETs (45). Several studies have suggested a new role for LL37 as a diagnostic or prognostic marker in various autoimmune diseases, such as systemic lupus erythematosus (SLE) and psoriatic arthritis (PsA) (9, 46). LL37 facilitates autoreactivity by stimulating plasmacytoid dendritic cells (pDCs) to produce type I interferons and acts as an autoantigen for pathogenic Th17 lymphocytes (46, 47). In SLE, LL37-specific T cells promoted B cell production of pathogenic anti-LL-37 autoantibodies. The work by Lande et al. also revealed the existence of specific T cells against citrullinated LL37. They were highly prominent in SLE and highly sporadic in psoriasis, implying that specificity against various autoantigens relies rather on disease-specific milieu than the nature of autoantigens (46). The increased levels of LL37 and PAD enzymes in the remission group imply the existence of a similar mechanism causing autoimmune reactions in patients with JIA.

Our study has several limitations. First, it demonstrates only a superficial view and provides only indirect evidence of the complicated processes of citrullination and NETosis in a cohort of patients with JIA; therefore, more detailed studies are needed. Second, the heterogeneity of our cohort could also serve as a limitation. Furthermore, detection of autoantibodies against anti-citrullinated proteins, such as citH3 or citLL37 could provide more information concerning their role in autoimmune disease.

Collectively, our data suggest that enhanced PTMs in JIA may result in the generation of anti-citrullinated/carbamylated autoantibodies against various epitopes later in life. This could contribute to ongoing inflammation associated with an autoimmunity, which can be ameliorated by regulating inflammation using immune therapy.

## Data availability statement

The raw data supporting the conclusions of this article will be made available by the authors, without undue reservation.

## Ethics statement

Written informed consent was obtained from all the patients or the patients' parents/guardians in accordance with the Declaration of Helsinki, and the study was approved by the Ethics Committee of University Hospital Motol. Written informed consent to participate in this study was provided by the participants' legal guardian/next of kin.

## Author contributions

ZP designed the study, performed experiments, analyzed data, and wrote the manuscript. IZ contributed to the data analysis and reviewed the manuscript. HM and DC provided biological material, and patient information and reviewed the manuscript. RH and AS provided human resources for the study, reviewed, and edited the manuscript. AS contributed to the funding acquisition and reviewed the manuscript. All authors contributed to the article and approved the submitted version.

## Funding

The study was supported by the Czech Ministry of Health AZV NU20-05-00320, by the institutional IPE2 funding of the Second Faculty of Medicine, Charles University, Prague and by the Ministry of Health, Czech Republic - conceptual development of research organization, Motol University Hospital, Prague, Czech Republic 00064203.

## Conflict of interest

The authors declare that the research was conducted in the absence of any commercial or financial relationships that could be construed as a potential conflict of interest.

## Publisher's note

All claims expressed in this article are solely those of the authors and do not necessarily represent those of their affiliated organizations, or those of the publisher, the editors and the reviewers. Any product that may be evaluated in this article, or claim that may be made by its manufacturer, is not guaranteed or endorsed by the publisher.

## References

[1] Mahmud SA Binstadt BA. Autoantibodies in the pathogenesis, diagnosis, and prognosis of juvenile idiopathic arthritis. Front Immunol. (2019) 10:3168. 10.3389/fimmu.2018.03168 30693002 PMC6339949

[2] Glerup M Rypdal V Arnstad ED Ekelund M Peltoniemi S Aalto K. Long-term outcomes in juvenile idiopathic arthritis: eighteen years of follow-up in the population-based nordic juvenile idiopathic arthritis cohort. Arth Care Res. (2020) 72:507–16. 10.1002/acr.23853 30762291

[3] Matsumoto T Matsui T Hirano F Tohma S Mori M. Disease activity, treatment and long-term prognosis of adult juvenile idiopathic arthritis patients compared with rheumatoid arthritis patients. Mod Rheumatol. (2020) 30:78–84. 10.1080/14397595.2018.1554228 30499364

[4] Shi J Van De Stadt LA Levarht EWN Huizinga TWJ Hamann D Van Schaardenburg D. Anti-carbamylated protein (anti-CarP) antibodies precede the onset of rheumatoid arthritis. Ann Rheum Dis. (2014) 73:780–3. 10.1136/annrheumdis-2013-204154 24336334

[5] Carubbi F Alunno A Gerli R Giacomelli R. Post-translational modifications of proteins: novel insights in the autoimmune response in rheumatoid arthritis. Cells. (2019) 8:657. 10.3390/cells8070657 31261953 PMC6678491

[6] Hissink Muller PCE Anink J Shi J Levarht EWN Reinards THCM Otten MH. Anticarbamylated protein (anti-CarP) antibodies are present in sera of juvenile idiopathic arthritis (JIA) patients. Ann Rheum Dis. (2013) 72:2053–5. 10.1136/annrheumdis-2013-203650 23873877

[7] Pratesi F Dioni I Tommasi C Alcaro MC Paolini I Barbetti F. Antibodies from patients with rheumatoid arthritis target citrullinated histone 4 contained in neutrophils extracellular traps. Ann Rheum Dis. (2014) 73:1414–22. 10.1136/annrheumdis-2012-202765 23727635

[8] Verheul MK Böhringer S van Delft MAM Jones JD Rigby WFC Gan RW. Triple positivity for anti-citrullinated protein autoantibodies, rheumatoid factor, and anti-carbamylated protein antibodies conferring high specificity for rheumatoid arthritis: implications for very early identification of at-risk individuals. Arthritis Rheumatol. (2018) 70:1721–31. 10.1002/art.40562 29781231

[9] Frasca L Palazzo R Chimenti MS Alivernini S Tolusso B Bui L. Anti-LL37 antibodies are present in psoriatic arthritis (PsA) patients: new biomarkers in PsA. Front Immunol. (2018) 9:1936. 10.3389/fimmu.2018.01936 30279686 PMC6154218

[10] Stoop JN Liu BS Shi J Jansen DTSL Hegen M Huizinga TWJ. Antibodies specific for carbamylated proteins precede the onset of clinical symptoms in mice with collagen induced arthritis. PLoS ONE. (2014) 9:102163. 10.1371/journal.pone.0102163 25025869 PMC4099068

[11] van Delft MAM Verheul MK Burgers LE Derksen VFAM van der Helm-van Mil AHM van der Woude D. The isotype and IgG subclass distribution of anti-carbamylated protein antibodies in rheumatoid arthritis patients. Arthritis Res Ther. (2017) 19:1–12. 10.1186/s13075-017-1392-z 28810902 PMC5558706

[12] Shi J Knevel R Suwannalai P Van Der Linden MP Janssen GMC Van Veelen PA. Autoantibodies recognizing carbamylated proteins are present in sera of patients with rheumatoid arthritis and predict joint damage. Proc Natl Acad Sci U S A. (2011) 108:17372–7. 10.1073/pnas.1114465108 21987802 PMC3198314

[13] Brinkmann V Reichard U Goosmann C Fauler B Uhlemann Y Weiss DS. Neutrophil extracellular traps kill bacteria. Science. (2004) 303:1532–5. 10.1126/science.1092385 15001782

[14] Saitoh T Komano J Saitoh Y Misawa T Takahama M Kozaki T. Neutrophil extracellular traps mediate a host defense response to human immunodeficiency virus-1. Cell Host Microbe. (2012) 12:109–16. 10.1016/j.chom.2012.05.015 22817992

[15] Urban CF Reichard U Brinkmann V Zychlinsky A. Neutrophil extracellular traps capture and kill Candida albicans yeast and hyphal forms. Cell Microbiol. (2006) 8:668–76. 10.1111/j.1462-5822.2005.00659.x 16548892

[16] Klocperk A Vcelakova J Vrabcova P Zentsova I Petruzelkova L Sumnik Z. Elevated biomarkers of NETosis in the serum of pediatric patients with type 1 diabetes and their first-degree relatives. Front Immunol. (2021) 12:699386. 10.3389/fimmu.2021.699386 34305937 PMC8293100

[17] Vecchio F Lo Buono N Stabilini A Nigi L Dufort MJ Geyer S. Abnormal neutrophil signature in the blood and pancreas of presymptomatic and symptomatic type 1 diabetes. JCI Insight. (2018) 3:122146. 10.1172/jci.insight.122146 30232284 PMC6237216

[18] Jariwala MP Laxer RM. NETosis in rheumatic diseases. Curr Rheumatol Rep. (2021) 23:1–12. 10.1007/s11926-020-00977-6 33511473

[19] Warnatsch A Ioannou M Wang Q Papayannopoulos V. Neutrophil extracellular traps license macrophages for cytokine production in atherosclerosis. Science. (2015) 349:316–20. 10.1126/science.aaa8064 26185250 PMC4854322

[20] Qiu S-L Zhang H Tang Q Bai J He Z-Y Zhang J-Q. Neutrophil extracellular traps induced by cigarette smoke activate plasmacytoid dendritic cells. Thorax. (2017) 72:1084–93. 10.1136/thoraxjnl-2016-209887 28720648

[21] Zentsova I Parackova Z Kayserova J Palova-Jelinkova L Vrabcova P Volfova N. Monocytes contribute to DNA sensing through the TBK1 signaling pathway in type 1 diabetes patients. J Autoimmun. (2019) 105:0–1. 10.1016/j.jaut.2019.06.005 31256920

[22] Parackova Z Zentsova I Vrabcova P Klocperk A Sumnik Z Pruhova S. Neutrophil extracellular trap induced dendritic cell activation leads to Th1 polarization in type 1 diabetes. Front Immunol. (2020) 11:661. 10.3389/fimmu.2020.00661 32346380 PMC7172866

[23] Khandpur R Carmona-Rivera C Vivekanandan-Giri A Gizinski A Yalavarthi S Knight JS. NETs are a source of citrullinated autoantigens and stimulate inflammatory responses in rheumatoid arthritis. Sci Transl Med. (2013) 5:178ra40–178ra40. 10.1126/scitranslmed.3005580 23536012 PMC3727661

[24] Sur Chowdhury C Giaglis S Walker UA Buser A Hahn S Hasler P. Enhanced neutrophil extracellular trap generation in rheumatoid arthritis: analysis of underlying signal transduction pathways and potential diagnostic utility. Arthritis Res Ther. (2014) 16:R122. 10.1186/ar4579 24928093 PMC4229860

[25] Corsiero E Pratesi F Prediletto E Bombardieri M Migliorini P. NETosis as source of autoantigens in rheumatoid arthritis. Front Immunol. (2016) 7:485. 10.3389/fimmu.2016.00485 27895639 PMC5108063

[26] Wang Y Li M Stadler S Correll S Li P Wang D. Histone hypercitrullination mediates chromatin decondensation and neutrophil extracellular trap formation. J Cell Biol. (2009) 184:205–13. 10.1083/jcb.200806072 19153223 PMC2654299

[27] Romero V Fert-Bober J Nigrovic PA Darrah E Haque UJ Lee DM. Immune-mediated pore-forming pathways induce cellular hypercitrullination and generate citrullinated autoantigens in rheumatoid arthritis. Sci Transl Med. (2013) 5: 209ra150. 10.1126/scitranslmed.3006869 24174326 PMC4032227

[28] Darrah E Rosen A Giles JT Andrade F. Peptidylarginine deiminase 2, 3 and 4 have distinct specificities against cellular substrates: novel insights into autoantigen selection in rheumatoid arthritis. Ann Rheum Dis. (2012) 71:92–8. 10.1136/ard.2011.151712 21859690 PMC3302156

[29] Zhou Y Chen B Mittereder N Chaerkady R Strain M An LL. Spontaneous secretion of the citrullination enzyme PAD2 and cell surface exposure of PAD4 by neutrophils. Front Immunol. (2017) 8:1200. 10.3389/fimmu.2017.01200 28993780 PMC5622307

[30] Consolaro A Ruperto N Bazso A Pistorio A Magni-Manzoni S Filocamo G. Development and validation of a composite disease activity score for juvenile idiopathic arthritis. Arthritis Rheum. (2009) 61:658–66. 10.1002/art.24516 19405003

[31] Perdomo J Leung HHL Ahmadi Z Yan F Chong JJH Passam FH. Neutrophil activation and NETosis are the major drivers of thrombosis in heparin-induced thrombocytopenia. Nat Commun. (2019) 10:1–14. 10.1038/s41467-019-09160-7 30899022 PMC6428879

[32] Holzer M Zangger K El-Gamal D Binder V Curcic S Konya V.. Myeloperoxidase-derived chlorinating species induce protein carbamylation through decomposition of thiocyanate and urea: novel pathways generating dysfunctional high-density lipoprotein. Antioxid. Redox Signal. (2012) 17:1043–52. 10.1089/ars.2011.4403 22462773 PMC3810648

[33] Tilvawala R Nguyen SH Maurais AJ Nemmara V V Nagar M Salinger AJ. The rheumatoid arthritis-associated citrullinome. Cell Chem Biol. (2018) 25:691. 10.1016/j.chembiol.2018.03.002 29628436 PMC6014894

[34] Van Venrooij WJ Pruijn GJM. How citrullination invaded rheumatoid arthritis research. Arthritis Res Ther. (2014) 16:1–5. 10.1186/ar4458 24472574 PMC4061769

[35] Ling S Cline EN Haug TS Fox DA Holoshitz J. Citrullinated calreticulin potentiates rheumatoid arthritis shared epitope signaling. Arthritis Rheum. (2013) 65:618–26. 10.1002/art.37814 23233327 PMC3582785

[36] Quirke A-M Fisher BAC Kinloch AJ Venables PJ Williams R Flügel A. Citrullination of autoantigens: Upstream of TNFα in the pathogenesis of rheumatoid arthritis. FEBS Lett. (2011) 585:3681–8. 10.1016/j.febslet.2011.06.006 21704035

[37] Andrade F Darrah E Gucek M Cole RN Rosen A Zhu X. Autocitrullination of human peptidyl arginine deiminase type 4 regulates protein citrullination during cell activation. Arthritis Rheum. (2010) 62:1630–40. 10.1002/art.27439 20201080 PMC2951335

[38] Sokolove J Zhao X Chandra PE Robinson WH. Immune complexes containing citrullinated fibrinogen costimulate macrophages via toll-like receptor 4 and Fcγ receptor. Arthritis Rheum. (2011) 63:53–62. 10.1002/art.30081 20954191 PMC3015008

[39] Sørensen OE Borregaard N. Neutrophil extracellular traps: the dark side of neutrophils. J Clin Invest. (2016) 126:1612–20. 10.1172/JCI84538 27135878 PMC4855925

[40] Thieblemont N Wright HL Edwards SW Witko-Sarsat V. Human neutrophils in auto-immunity. Semin Immunol. (2016) 28:159–73. 10.1016/j.smim.2016.03.004 27036091

[41] Hu X Xie Q Mo X Jin Y. The role of extracellular histones in systemic-onset juvenile idiopathic arthritis. Ital J Pediatr. (2019) 45:1–9. 10.1186/s13052-019-0605-2 30642364 PMC6332604

[42] Knight JS Carmona-Rivera C Kaplan MJ. Proteins derived from neutrophil extracellular traps may serve as self-antigens and mediate organ damage in autoimmune diseases. Front Immunol. (2012) 3:380. 10.3389/fimmu.2012.00380 23248629 PMC3521997

[43] Lewis HD Liddle J Coote JE Atkinson SJ Barker MD Bax BD. Inhibition of PAD4 activity is sufficient to disrupt mouse and human NET formation. Nat Chem Biol. (2015) 11:189–91. 10.1038/nchembio.1735 25622091 PMC4397581

[44] Darrah E Giles JT Ols ML Bull HG Andrade F Rosen A. Erosive rheumatoid arthritis is associated with antibodies that activate PAD4 by increasing calcium sensitivity. Sci Transl Med. (2013) 5: 186ra65–186ra65. 10.1126/scitranslmed.3005370 23698378 PMC3740946

[45] Neumann A Berends ETM Nerlich A Molhoek EM Gallo RL Meerloo T. The antimicrobial peptide LL-37 facilitates the formation of neutrophil extracellular traps. Biochem J. (2014) 464:3–11. 10.1042/BJ20140778 25181554

[46] Lande R Palazzo R Gestermann N Jandus C Falchi M Spadaro F. Native/citrullinated LL37-specific T-cells help autoantibody production in systemic lupus erythematosus. Sci Rep. (2020) 10:1–14. 10.1038/s41598-020-62480-3 32245990 PMC7125190

[47] Lande R Gregorio J Facchinetti V Chatterjee B Wang Y-H Homey B. Plasmacytoid dendritic cells sense self-DNA coupled with antimicrobial peptide. Nature. (2007) 449:564–9. 10.1038/nature06116 17873860

