# Supplementary material for: Increased histone citrullination in juvenile idiopathic arthritis
Source: Front Med (Lausanne). 2022 Aug 19;9:971121. doi: 10.3389/fmed.2022.971121 (PMC9437311; doi:10.3389/fmed.2022.971121)
Supplement: Supplementary file 1 [file Table_1.docx]

Increased histone citrullination in juvenile idiopathic arthritis

Zuzana Parackova^1^*, Irena Zentsova^1^, Hana Malcova^2^, Dita Cebecauerova^2^, Anna Sediva^1^, Rudolf Horvath^2^

^1^Department of Immunology, 2^nd^ Faculty of Medicine, Charles University, University Hospital Motol, Prague, Czech Republic

^2^ Department of Paediatric and Adult Rheumatology, University Hospital Motol, Prague, Czech Republic

***Correspondence:**

Zuzana Parackova, Department of Immunology, 2^nd^ Faculty of Medicine, Charles University, University Hospital Motol, V Uvalu 84, Prague 5, 15006, +420224435971, [zuzana.parackova@fnmotol.cz](mailto:zuzana.parackova@fnmotol.cz)

Supplementary Table 1: Cohort characteristics

| COHORT | **ALL (n=30)** | **Active (n=20)** | **Remission (n=10)** |
| --- | --- | --- | --- |
| Demography | | | |
| Gender (males, %) | 10 (33·3%) | 6 (30%) | 4 (40%) |
| Age (mean, +/-SD, yr·) | 13·06±3·71 | 12·46±4·11 | 14·9±2·78 |
| Disease characteristics | | | |
| Oligoarticular/Oligoarticular-extended | 10 (33·3%) | 6 (30%) | 4 (40%) |
| Polyarticular | 9 (30%) | 6 (30%) | 3 (30%) |
| ERA | 11 (36·67%) | 8 (26·67%) | 3 (30%) |
| ANA (n, %) | 14 (46·67%) | 9 (30%) | 5 (50%) |
| HLA-B27 (n, %) | 11 (36·67%) | 9 (30%) | 2 (20%) |
| RF positivity (n, %) | 1 (3·33%) | 1 (5%) | 0 (0%) |
| ACPA positivity (n, %) | 0 (0%) | 0 (0%) | 0 (0%) |
| Disease activity | | | |
| active joint count (mean +/- SD) | 3·53±3,47 | 5·3±2·92 | 0 |
| active uveitis (n) | 3 (10%) | 3 (15%) | 0 (0%) |
| ESR (mean +/- SD, mm/hour) | 10·48±7·23 | 12·58±7·67 | 6·5±4·27 |
| CRP (mean +/- SD, mG/L) | 6·42±11·96 | 9·39±13·98 | 0·79±0·81 |
| JADAS71 | 8·43±7·26 | 12·65±4·94 | 0 |
| Treatment |  |  |  |
| GC per.os (n, %) | 8 (26·6%) | 4 (20%) | 0 (0%) |
| csDMARDs (n, %) | 30 (100%) | 20 (100%) | 10 (100%) |
| anti-TNFa (n,%) | 10 (33·3%) | 0 (0%) | 10 (100%) |

Baseline characteristics of the study cohort: 30 enrolled patients with juvenile idiopathic arthritis (JIA) with active disease (JADAS71≥1) and in remission (JADAS71<1 lasting for at least 6 months)·JIA active patients consequently underwent therapy with TNF inhibitors. Experiments were performed before the therapy initiation. (SD= standard deviation, mo·= months, yr-= years, ERA= Enthesitis-related Arthritis, ANA= Antinuclear Antibodies, HLA-B27= Human Leukocyte Antigen B27, RF= Rheumatoid Factor, ACPA= Anti-citrullinated Protein Antibodies, ESR= Erythrocyte sedimentation rate, CRP= C-reactive protein,P JADAS71= Juvenile Arthritis Disease Activity Score, GC= Glucocorticoids, csDMARDS= Conventional Synthetic Disease-Modifying Antirheumatic Drugs, anti-TNFa= anti-Tumor Necrosis Factor-alpha)·
